# Anticancer activity of *7-epiclusianone*, a benzophenone from *Garcinia brasiliensis*, in glioblastoma

**DOI:** 10.1186/s12906-015-0911-1

**Published:** 2015-10-30

**Authors:** Leilane Sales, Julia Alejandra Pezuk, Kleiton Silva Borges, María Sol Brassesco, Carlos Alberto Scrideli, Luiz Gonzaga Tone, Marcelo Henrique dos Santos, Marisa Ionta, Jaqueline Carvalho de Oliveira

**Affiliations:** Institute of Natural Sciences, Federal University of Alfenas, Rua Gabriel Monteiro da Silva, 700, 37130-000 Alfenas, MG Brazil; Molecular Oncology Center, Sirio Libanes Hospital, Rua Dona Adma Jafet, 115, 01308-050 Sao Paulo, SP Brazil; Department of Paediatrics, Ribeirão Preto Medical School, University of São Paulo, Av. Bandeirantes, 3900, 14049-900 Ribeirão Preto, SP Brazil; Department of Biology, Faculty of Philosophy, Sciences and Letters at Ribeirão Preto, University of São Paulo, Av. Bandeirantes, 3900, 14049-900 Ribeirão Preto, SP Brazil; Institute of Chemistry, Federal University of Viçosa, Avenida Peter Henry Rolfs, s/n, 36570-900 Viçosa, MG Brazil; Institute of Biomedical Sciences, Federal University of Alfenas, Rua Gabriel Monteiro da Silva, 700, 37130-000 Alfenas, MG Brazil

**Keywords:** Glioblastoma, 7-*epiclusianone*, Viability, Apoptosis, Cell cycle

## Abstract

**Background:**

Glioblastoma is the most common tumor of the central nervous system and one of the hardest tumors to treat. Consequently, the search for novel therapeutic options is imperative. 7-*epiclusianone*, a tetraprenylated benzophenone isolated from the epicarp of the native plant *Garcinia brasiliensis*, exhibits a range of biological activities but its prospect anticancer activity is underexplored. Thus, the aim of the present study was to evaluate the influence of 7-*epiclusianone* on proliferation, clonogenic capacity, cell cycle progression and induction of apoptosis in two glioblastoma cell lines (U251MG and U138MG).

**Methods:**

Cell viability was measured by the MTS assay; for the clonogenic assay, colonies were stained with Giemsa and counted by direct visual inspection; For cell cycle analysis, cells were stained with propidium iodide and analyzed by cytometry; Cyclin A expression was determined by immunoblotting; Apoptotic cell death was determined by annexin V fluorescein isothiocyanate labeling and Caspase-3 activity in living cells.

**Results:**

Viability of both cell lines was drastically inhibited; moreover, the colony formation capacity was significantly reduced, demonstrating long-term effects even after removal of the drug. 7-*epiclusianone* treatment at low concentrations also altered cell cycle progression, decreased the S and G2/M populations and at higher concentrations increased the number of cells at sub-G1, in concordance with the increase of apoptotic cells.

**Conclusion:**

The present study demonstrates for the first time the anticancer potential of 7-*epiclusianone* against glioblastoma cells, thus meriting its further investigation as a potential therapeutic agent.

## Background

Glioblastoma (GBM) is the most aggressive and common glial tumors, and it represents the main type of primary cancer of the central nervous system in adults [1]. The actual standard treatment for GBM combines tumor resection, chemotherapy with temozolomide and radiotherapy [2]. However, despite management improvements, the outcome of patients remains extremely poor, with a mean life expectancy of approximately one year and a 2-year overall survival of only 15.4 % [3]. Therefore, GBM remains as one of the hardest tumors to treat, making search for novel therapeutic options imperious.

Historically, natural products have been the foundation for the development of medical compounds and approximately 50 % of anticancer drugs are still natural products or their derivatives [4, 5]. Brazilian biodiversity offers many examples of common and rare plant life that deserves to be better explored. Among the variety of plant extracts described so far, 7-*epiclusianone*, a tetraprenylated benzophenone isolated from the epicarp of the native plant *Garcinia brasiliensis* (commonly known as *bacupari*), has traditionally been used in the folk medicine for the treatment of different diseases [6].

*7-epiclusianone* has shown important biological effects including anti-inflammatory [7, 8], antinociceptive [7, 9], antimicrobial [10, 11], antispasmodic [12], antianaphylactic [13] and antiprotozoal activities [14]. On the other hand, the potential anticancer and apoptotic effect is underexplored.

Based on such findings, the present study points 7-epi*clusianone* as a potential antineoplastic compound, demonstrating its influence on growth, cell cycle dynamics, apoptosis and colony formation ability against glioblastoma cells.

## Methods

### Cell culture conditions

The adult human GBM cell lines U138MG (p53^mut^, PTEN^mut^, p14ARF/p16^del^, high MGMT levels) and U251MG (p53^wt^, PTEN^null^, p14ARF/p16^del^, low MGMT levels) were purchased from the American Type Culture Collection, USA. Cell cultures were maintained in DMEM (Dulbecco’s Modified Eagle’s Minimum Essential Medium, Sigma, CA, USA) supplemented with 10 % fetal bovine serum (FBS, Cultilab, Sao Paulo, Brazil) and grown in a 37 °C humidified incubator containing 5 % CO_2_.

### Isolation and characterization of 7-*epiclusianone*

The fruits of *Garcinia brasiliensis*, after identification of plant material by Dr. João Augusto Alves Meira Neto, were collected from trees growing under controlled conditions at the herbarium of the Federal University of Viçosa – UFV (latitude 20°45′14″ south and longitude 42°52′55″ west), Minas Gerais state, Brazil, where the voucher specimen (#VIC2604) is deposited.

**Fig. 1:**
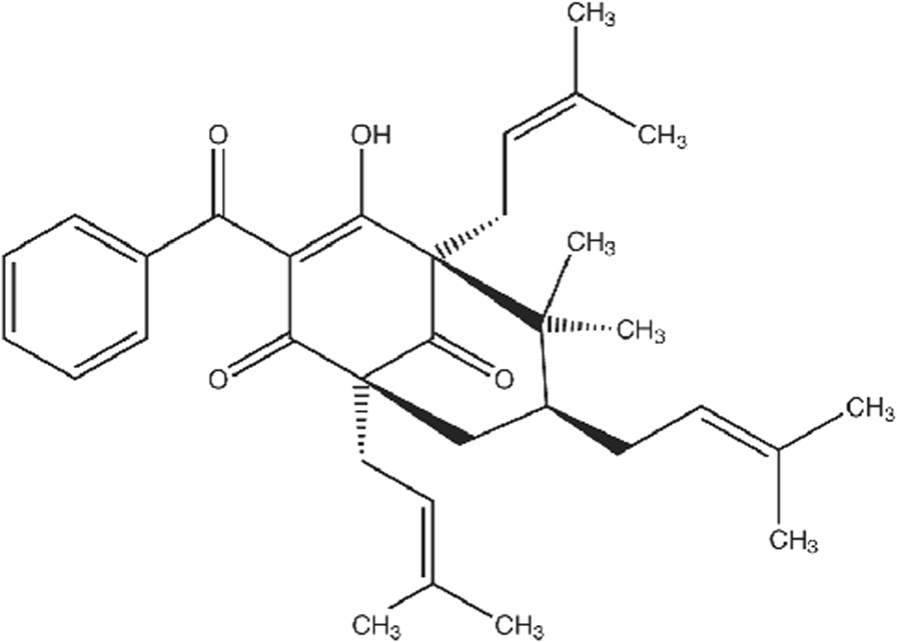
Chemical structure of 7-*epiclusianone*

To obtain the extract, 1000 g of *G. brasiliensis* fruit epicarps were dried at 40 °C in a forced air oven for 48 h. Dried and powdered epicarps were milled and extracted by maceration using EtOH (3 L) during 24 h. Evaporation of the solvent under reduced pressure at 45 °C afforded the crude EtOH extract, which was chromatographed on silica gel (230–400 mesh) column and eluted with crescent polarity mixtures of (n-hexane, n-hexane-ethyl acetate (95:5), n-hexane-ethyl acetate (80:20), n-hexane-ethyl acetate (50:50), n-hexane-ethyl acetate (20:80) and ethanol) to give 25 fractions. These fractions were pooled into four groups according to their similarities after the analysis using thin layer chromatography (TLC) and compared to the standard 7-*epiclusianone* previously isolated. Fractions 4–10 were chromatographed on a silica gel (230–400 mesh) column (8 × 100 cm) eluted with the same crescent polarity mixtures of n-hexane/ethyl-acetate and ethyl-acetate/ethanol to purify the prenylated benzophenone 7-epiclusianone with a yield of 5 %. Its structure (Fig. 1) was identified as the polyprenylated benzophenone 7-*epiclusianone* [(1R,5R,7R)-3-benzoyl-4-hydroxy-8,8-dimethyl-1,5,7-tris(3-methylbut-2-en-1-yl) bicyclo[3.3.1]non-3-ene-2,9-dione] using infrared, ultraviolet, and mass spectra data and nuclear magnetic resonance spectroscopy, and its molecular geometries established using single crystal X-ray diffraction (XRD) analysis. The purity level was > 99.85 % as determined by HPLC [15, 16].

For *in vitro* testing, 7-*epiclusianone* was dissolved in dimethyl sulfoxide (DMSO), and this stock solution was diluted in fresh medium at the appropriated concentrations immediately before of use. Vehicle alone was used as control and the final concentration of the solvent did not exceed 0.5 % (v/v).

### Cell viability

Cells were plated on 96-wells at 5×10^3^. After attachment (24 h), the cells were treated for 48 h with 7-*epiclusianone* at different concentrations (10 μM, 20 μM, 30 μM or 40 μM). The Promega® non-radioactive cell proliferation assay was used to determinate cell viability. This assay measures the amount of formazan produced from [3-(4,5-dimethylthiazol-2-yl)-5-(3-carboxymethoxyphenyl)-2-(4-sulfophenyl)-2H-tetrazolium, inner salt, MTS)] by the dehydrogenase enzymes of metabolically active cells (Promega, Madison, WI, USA). Thus, the quantity of formazan produced (as measured by the absorbance at 490 nm) is directly proportional to the number of living cells. Absorbance values of the treated cells were compared with the absorbance values of untreated cells. The experiments were conducted in triplicate wells and repeated twice. Means ± standard deviations (SD) were calculated. The IC_50_ value was determined from non-linear regression using GraphPad Prism® (GraphPad Software, Inc., San Diego, CA, USA).

### Colony formation assay

Clonogenic formation assay was performed according to Franken et al. [17]. Two hundred cells were seeded in 35 mm plates. After 24 h, cells were treated with 7-*epiclusianone* for 8 h and subsequently were incubated for 10 days with drug-free medium. After that period, colonies were fixed and stained with Giemsa (3 %). Only colonies with >50 cells were counted by direct visual inspection with a stereo microscope at 20× magnification. Assays were performed in triplicate.

### Cell cycle analysis

For cell cycle analysis, 2 × 10^5^ cells were seeded on 25 cm^2^ flasks and treated for 48 h. Cells were then trypsinized and fixed in 70 % ethanol, stained with propidium iodide, and analyzed with a Guava Personal Cell Analysis system (Guava Technologies, Hayward, CA, USA) according to the protocol provided by the manufacturer. Percentages of cells in sub-G1, G0/G1, S, or G2/M phase were determined and processed using the GUAVA Cytosoft software, version 4.2.1. Assays were performed three times on separate occasions.

### Immunoblot

Both cell lines were treated with 10 μM and 40 μM for 48 h. Cells were then lysed in lysis buffer (RIPA; Sigma Chemical Co.; 10 % protease, and 10 % phosphatase) and stored at – 80 °C until use.

Anti-Cyclin-A (sc-751, 1:1000) and anti-GAPDH (sc-47724, 1:1000) antibodies were purchased from Santa Cruz Biotechnology (Santa Cruz, CA, USA). Equal amounts of protein (40 mg) were size-fractionated by 12 % SDS-PAGE, blotted onto a nitrocellulose membrane (Amersham Hybond™ ECL™, GE Healthcare) and incubated in Tris-buffered saline-0.1 % Tween-20 (TBS-T) containing 5 % (w/v) dried non-fat milk for 1 h at room temperature. After blocking and washing in TBS-T with 0.1 % Tween 20 for 30 min, each membrane was incubated overnight with appropriately diluted primary antibodies. After incubation, the membrane was washed three times in TPBS-T with 0.1 % Tween 20 and bound to a biotin-labeled horseradish peroxidase–conjugated species-specific secondary antibody (AbCam, San Francisco, CA, USA). The complexes were visualized using an enhanced chemiluminescence reagent (ECL; Amersham, Uppsala, Sweden).

### Apoptosis assay

Apoptotic cell death was determined by annexin V fluorescein isothiocyanate labeling (BD Biosciences Pharmigen, San Jose, CA, USA). Briefly, cells were grown in 25 cm^2^ tissue culture flasks and treated with *7-epiclusianone* for 48 h cells were then trypsinized and centrifuged at 1,000 rpm for 5 min at 4 °C, washed with ice-cold PBS, and then 2 × 10^5^ cells were resuspended in 300 μl of 1× annexin V binding buffer (BD Biosciences Pharmigen). Cells were stained with 5 μl annexin V fluorescein isothiocyanate and 50 μl of propidium iodide (50 μmol/L), and immediately read. At least 10,000 events were analyzed using a BD FACSCalibur flow cytometer (BD Biosciences Pharmigen). Assays were performed in triplicate.

Apoptotic cell death was also determined by analysis of caspase activation. 5 × 10^4^ cells were seeded on 6-well plates containing 3 mL of culture medium. After 24 h, the medium was replaced and cells treated with 40 μM of *7-epiclusianone* or vehicle only and cultured for additional 48 h. Caspase activation was determined using the NucView 488 Caspase-3 Detection in Living Cells kit (Biotium Inc. #30029) according to the manufacturer’s instructions. Five hundred nuclei per treatment were analyzed by fluorescence microscopy.

### Statistical analysis

Statistical analyses were performed using the SigmaStat 3.5 software (Jandel Scientific Company, San Rafael, CA, USA). One-way repeated measures analysis of variance followed by the Holm–Sidak pairwise multiple comparison were used to establish any significant differences between groups. For caspase activation analysis, Student’s *t*-test was used. All tests were carried out for α = 0.05.

## Results and discussion

**Fig. 2:**
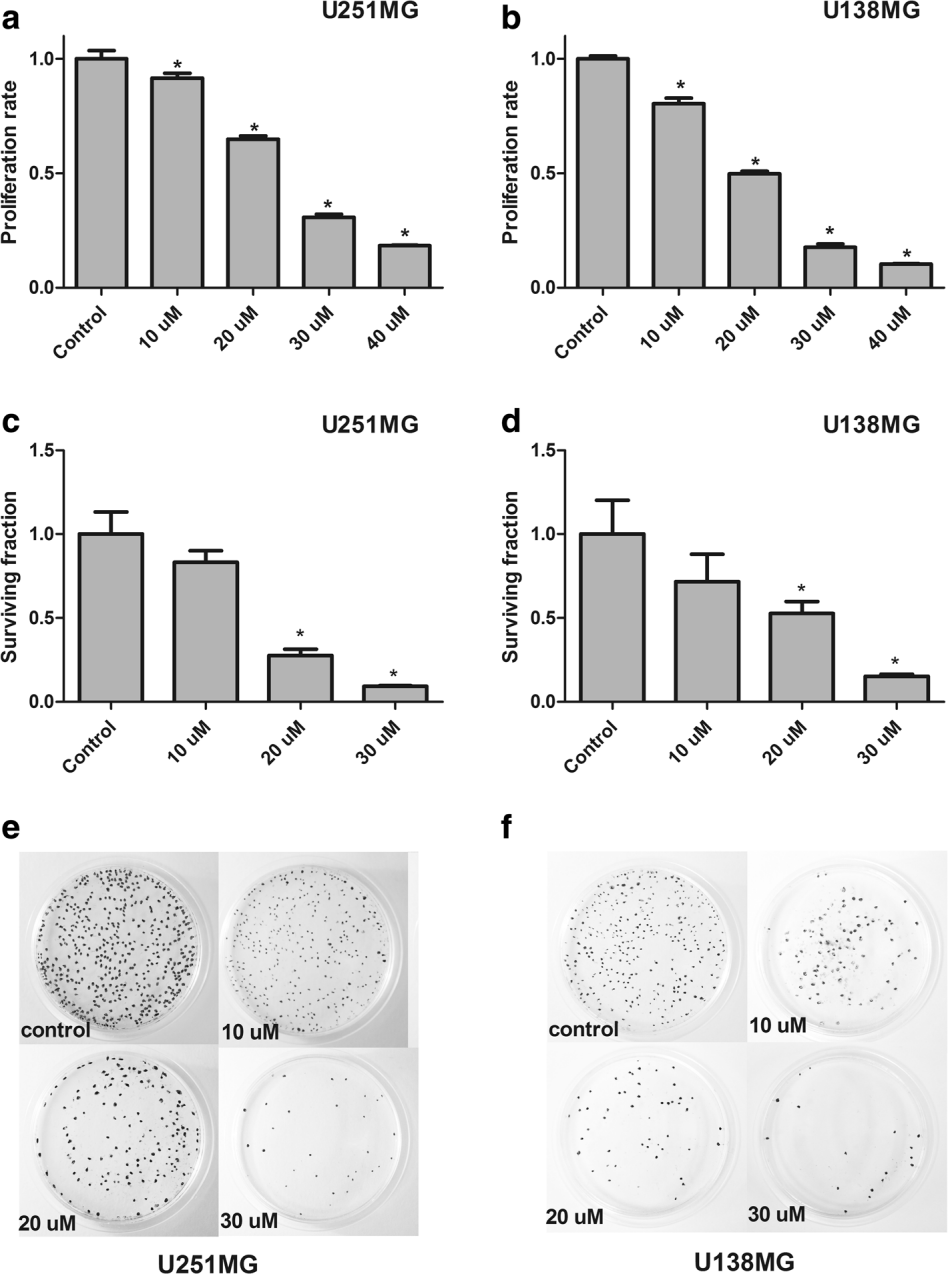
7-*epiclusianone* treatments inhibit growth and clonogenic capacity. **a**, **b** Growth inhibition in U251MG and U138MG cell lines treated with 7-*epiclusianone* at the indicated concentrations for 48 h (MTS assay); (**c**-**f**) 7-*epiclusianone* potently abrogated the clonogenic capacity of both glioblastoma cell lines (*p < 0.01)

7-*epiclusianone* significantly inhibited cell viability in both GBM lines tested when compared to control in a concentration-dependent manner (p < 0.01) (Fig. 2a and b). The concentration required to induce 50 % inhibition of cell growth (IC_50_) after 48 h of treatment was 23.00 ± 0.32 μM in U251MG (r^2^ = 0.99) and 18.52 ± 0.50 μM (r^2^ = 0.98) in U138MG.

7-*epiclusianone* treatment also reduced the colony formation capacity for both cell GBM lines when compared to control at 20 μM and 30 μM (*p* < 0.01), demonstrating long-term effects even after removal of the drug (Fig. 2c-f).

**Fig. 3:**
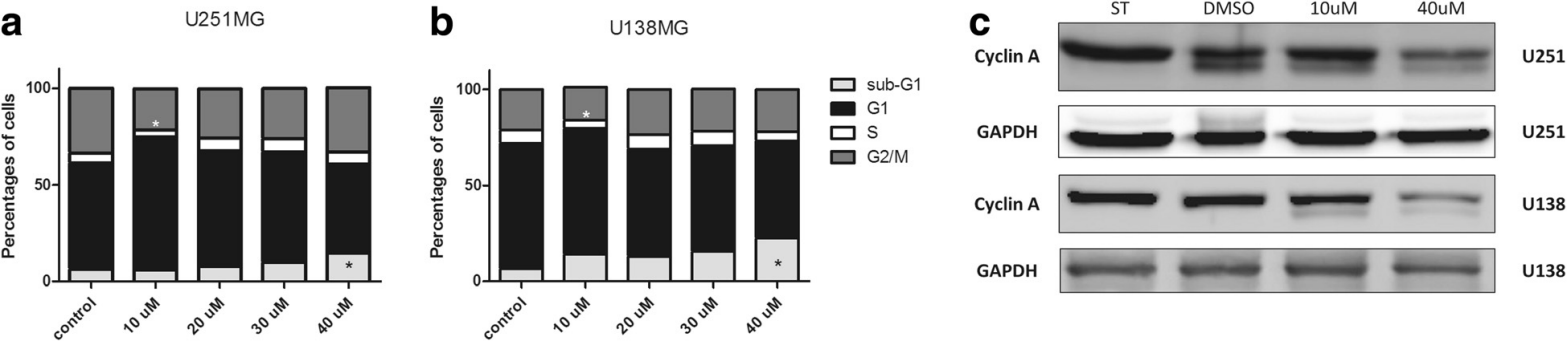
7-*epiclusianone* treatments alter cell cycle progression. Cell cycle progression in U251MG (**a**) and U138MG (**b**) glioblastoma cell lines after 48 h; **c** Cyclin A and GAPDH expression after 48 h. *p < 0.01

Cell cycle analysis showed that 7-*epiclusianone* is effective in inducing cell cycle arrest and/or cell death, however these effects are concentration-dependent. Increased G1 population with concomitant decreased G2/M populations was observed in U251MG cultures treated with 7-*epiclusianone* at 10 μM, in U138MG there were also decrease in S and G2/M populations. When higher concentration of 7-*epiclusianone* was used (40 μM), a significant increase in Sub-G1 population was observed for both cell lines (Fig. 3a and b, Table 1).

**Table 1:** Cell cycle analysis of glioblastoma cell lines after treatment with 7-*epiclusianone*

	U251MG		U138MG	
Sub-G1 (%)				
Control	6.35	±0.92	6.45	±3.68
10 μM	5.92	±3.00	13.98	±6.14
20 μM	7.80	±1.00	12.99	±0.81
30 μM	9.88	±1.09	15.66	±2.82
40 μM	14.74	±0.94*	22.50	±2.45*
G1 (%)				
Control	54.97	±2.76	65.54	±3.86
10 μM	68.94	±2.34*	65.70	±4.20
20 μM	59.67	±3.82	55.99	±0.54*
30 μM	57.02	±2.97	54.92	±0.68*
40 μM	45.77	±1.49*	50.72	±2.58*
S (%)				
Control	5.07	±1.07	6.83	±0.73
10 μM	3.59	±1.63	4.32	±0.76*
20 μM	6.77	±0.41	7.44	±0.75
30 μM	6.93	±0.39	7.45	±0.92
40 μM	6.23	±1.92	4.74	±0.17*
G2/M (%)				
Control	33.79	±2.34	21.24	±0.50
10 μM	21.36	±1.05*	17.25	±3.05*
20 μM	25.44	±2.99*	23.51	±0.90
30 μM	26.10	±1.66*	22.32	±3.65
40 μM	33.62	±2.28	21.99	±1.92

Percentages of cells in Sub-G1, G1, S and G2/M phases are expressed as mean ± standard deviation

*p < 0.05

Cell cycle arrest was accompanied by decreased CCNA (cyclin A) expression (Fig. 3c), which is a cell cycle regulatory protein repressed during G1 phase and induced at S-phase [18]. Furthermore, ectopically expressed cyclin A overcomes G1 arrest *in vitro* [19], suggesting that 7-*epiclusianone* induced G1-phase arrest is at least partially caused by cyclin A down regulation.

**Fig. 4:**
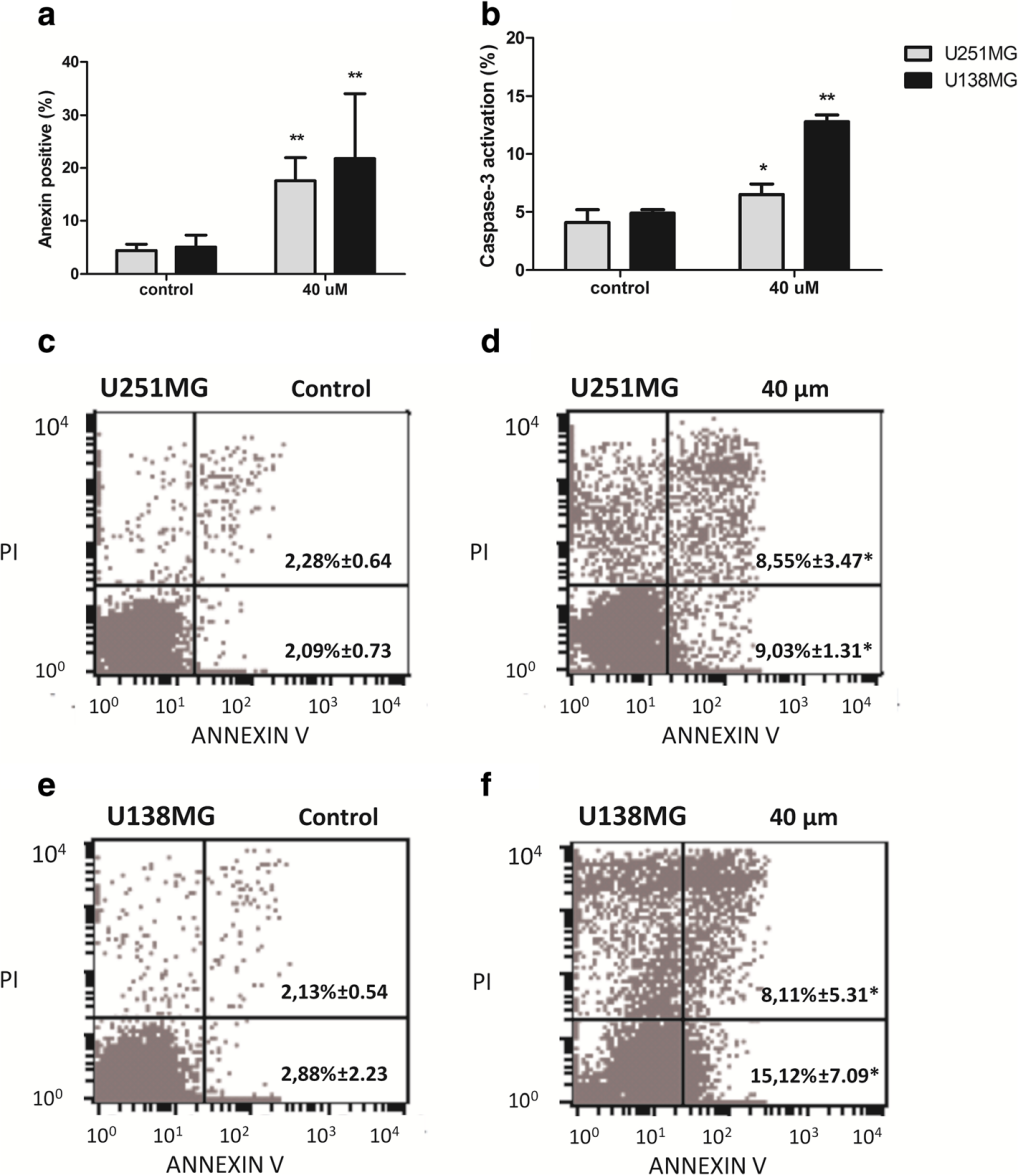
7-*epiclusianone* treatments alter apoptotic rates. **a**, **c**-**f** Increased Annexin V positive cells after treatment with 7-*epi*-clusianone in glioblastoma (GBM) cells lines; **b** Increased caspase-3 activation rates after treatment with 7-*epiclusianone* in GBM cells lines (data are expressed as the mean-SD of all cell lines). *p < 0.05, **p < 0.01. PI, propidium iodide

Besides G1 arrest, after high dose treatment (40 μM) cell cycle analysis showed increase in the sub-G1 population. Considering the results obtained, we also investigated whether cell death detected would be due to apoptosis induction. Annexin V was used for monitoring cells in apoptosis and the 40 μM treatment mediated a significant increase of positive cells to 18.8 % for U251MG and to 21.8 % for U138MG (*p* < 0.01) after 48 h (Fig. 4a and c-f). This result was also confirmed by increased level of cell caspase-3 activity (Fig. 4b). Additionally, the ratio of proliferation and apoptosis (annexin V positive cells) in GBM cells were 22.83 (U251MG) and 19.92 (U138MG) before treatment, after 40 μM 7-epiclusianone exposition, the ratio strongly decreased for 1.05 in U251MG and 0.48 in U138MG cell lines.

The Clusiaceae family is frequently found in tropical areas and comprises about 40 genera and 1200 species, with about 400 species of *Garcinia* genius [10]. Several benzophenones extracted from *Garcinia* spp. such as *G. indica, G. xanthochymus, G. purpurea, G. macrophylla, G. mangostana, G. pyrifera,* and *G. yunnanensis* have previously exhibited cytotoxic activity against various tumor types, including prostate, ovarian, colon, leukemia and cervical cancer [20–22].

*Garcinia brasiliensis* fruits have long been used as an alternative treatment in Brazilian folk medicine. In the present study, we showed that its derivative, 7-*epiclusianone*, diminishes cell viability, alters cell cycle progression, and increases apoptosis in GBM, one of the most aggressive tumors and with limited treatment options.

So far, few articles suggested a potential antiproliferative effect of *G. brasiliensis* after 7-*epiclusianone* treatment of human cancer cell lines, including melanoma, kidney, ovarian, prostate and lung [23, 24].

In A549 lung cells, 7-*epiclusianone* treatment also induced apoptosis and cell cycle arrest in G1/S transition. Additionally, the cytotoxic activity of 7-*epiclusianone* in normal fibroblasts (CCD-1059Sk) was also evaluated and IC_50_ value was approximately 58 μM, 2.5 and 3.2 times higher than for the cancer cell lines tested herein, U251MG and U138MG [24].

Similar cytotoxic activities have been described for another polyisoprenylated benzophenone isolated and purified from *G. xanthochymus* which arrests the cell-cycle in G1-phase and induces apoptosis in colon cancer cell lines [25]. Apoptosis has also been evidenced after treatment with benzophenones extracted from *G. xanthochymus* [26], from *G. yunnanensis* [27], and from *G. purpurea* [28] in many solid tumors and leukemia cells. Comparatively, Garcinol (obtained from *G. indica*), the most cited natural benzophenone in the literature, has repeatedly demonstrated to decrease cell viability and induce apoptosis of cancer cells [29, 30]. This compound also decreased the colony forming ability of prostate and pancreatic cancer cell lines, suggesting a possible application against metastatic disease [31].

It is well known that GBM treatment outcome is often hampered by the inherent invasive properties of the tumor, thus the clonogenic capacity of cells after treatment is an important aspect for testing potential therapeutic compounds. Herein, 7-*epiclusianone* treatment for only 8 h significantly reduced the colony formation capacity of GBM cells, demonstrating long-term effects even after removal of the drug.

Another benzophenone derived from the Malaysian *G. hombroniana,* 2,3′,4,5’-tetrahydroxy-6-methoxybenzophenone, has also presented cytotoxic effects on the DBTRG cell line which is derived from a 56-year-old woman with GBM. Most interestingly, the compound was found to be more toxic towards glioma cells as compared to other cell lines of different origin including MCF-7 (breast cancer), U2OS (osteosarcoma) and PC-3 (prostate cancer) [32].

In the present study, we investigated 7-*epiclusianone* in glioma cells mainly due to extremely poor outcome of patients caused for few chemotherapy options available. However, the numerous antitumor effects described for other natural benzophenone recommend future investigation of 7-*epiclusianone* also in other tumor types.

Even though the mechanisms through which benzophenones impair cell growth and induce apoptosis are still under investigation, some studies suggest an anti-microtubule action during cell division or the intensification of caspase-3 activity [25, 28, 29]. Other mechanisms such as inhibition of histone acetyltranferase activity [33], inhibition of kinase activity [34], or inducing DNA damage [35] have also been suggested.

## Conclusions

The results presented herein point 7-*epiclusianone* as an important natural benzophenone with antineoplastic activity in a glioblastoma model, a tumor with inherent chemoresistance, demonstrating influence on cell growth, cell cycle dynamics, apoptosis and colony formation ability. However, further studies should be conducted to investigate the molecular mechanisms underneath the cytotoxic activity of this benzophenone, pharmacokinetics in human condition and the prospect of using it for the treatment of other tumor types.
